# Perception of emotionally incongruent cues: evidence for overreliance on body vs. face expressions in Parkinson's disease

**DOI:** 10.3389/fpsyg.2024.1287952

**Published:** 2024-05-06

**Authors:** Yasmin Abo Foul, David Arkadir, Anastasia Demikhovskaya, Yehuda Noyman, Eduard Linetsky, Muneer Abu Snineh, Hillel Aviezer, Renana Eitan

**Affiliations:** ^1^Department of Psychology, Hebrew University of Jerusalem, Jerusalem, Israel; ^2^Brain Division, Hadassah-Hebrew University Medical Center, Jerusalem, Israel; ^3^Neuropsychiatry Unit, Jerusalem Mental Health Center, Hebrew University of Jerusalem, Jerusalem, Israel; ^4^Department of Medical Neurobiology (Physiology), Institute for Medical Research Israel-Canada, Hebrew University-Hadassah Medical School, Jerusalem, Israel; ^5^Department of Psychiatry, Brigham and Women's Hospital, Harvard Medical School, Boston, MA, United States

**Keywords:** emotional integration, Parkinson's disease, body language, context, schizophrenia, emotional perception

## Abstract

Individuals with Parkinson's disease (PD) may exhibit impaired emotion perception. However, research demonstrating this decline has been based almost entirely on the recognition of isolated emotional cues. In real life, emotional cues such as expressive faces are typically encountered alongside expressive bodies. The current study investigated emotion perception in individuals with PD (*n* = 37) using emotionally incongruent composite displays of facial and body expressions, as well as isolated face and body expressions, and congruent composite displays as a baseline. In addition to a group of healthy controls (HC) (*n* = 50), we also included control individuals with schizophrenia (SZ) (*n* = 30), who display, as in PD, similar motor symptomology and decreased emotion perception abilities. The results show that individuals with PD showed an increased tendency to categorize incongruent face-body combinations in line with the body emotion, whereas those with HC showed a tendency to classify them in line with the facial emotion. No consistent pattern for prioritizing the face or body was found in individuals with SZ. These results were not explained by the emotional recognition of the isolated cues, cognitive status, depression, or motor symptoms of individuals with PD and SZ. As real-life expressions may include inconsistent cues in the body and face, these findings may have implications for the way individuals with PD and SZ interpret the emotions of others.

## 1 Introduction

Emotion perception, a vital skill for successful social interactions, may be affected by neurological disease, psychiatric disorders, or normal aging of the brain (Phillips et al., 2003; Hayes et al., 2020). Such is the case in PD, a neurodegenerative disorder caused by the breakdown and death of the dopamine-secreting neurons of the substantia nigra pars compacta (for a review, see Argaud et al., 2018). The hallmark manifestation of PD involves motor symptoms such as muscular rigidity, bradykinesia (slowness of movement), tremor, and decreased facial expressivity known as “masked face” (hypomimia), which manifest in the early stages of PD (Gelb et al., 1999; Assogna et al., 2008). Individuals with PD may also display significant non-motor symptoms, such as cognitive decline (Hindle et al., 2014), psychiatric symptoms (Schneider et al., 2008), and difficulties in social interactions, among other systemic symptoms. These non-motor manifestations dramatically impact their quality of life (Bernal-Pacheco et al., 2012).

### 1.1 Emotion perception difficulties in PD

One important class of difficulties in PD involves a wide range of emotion perception deficits, appearing in the early stages of the disease and deteriorating with its progression (Lin et al., 2016). These latter symptoms include impaired production and perception of emotional prosody (Schröder et al., 2010), impaired discrimination of affective speech (Pell and Leonard, 2003), as well as impaired production and perception of facial expressions (for a meta-analysis, see Jacobs et al., 1995; Simons et al., 2004; Gray and Tickle-Degnen, 2010; Gunnery et al., 2016). Recent meta-analyses and reviews have shown that individuals with PD have a generalized deficit in emotion perception across modalities, which is more pronounced for negative emotional expressions, specifically anger, fear, sadness, and disgust (Argaud et al., 2018; Coundouris et al., 2019; Gothwal et al., 2022). While these overall findings are important, specific results differ across studies, perhaps due to significant variance with regard to participants' characteristics (e.g., the PD stage, cognitive abilities) and experimental tasks and design. As part of this variance, Argaud et al. (2018) noted that roughly a third of the studies found no difference in facial expression perception between individuals with PD and controls.

### 1.2 Suggested underlying mechanism

The specific mechanisms underlying these difficulties in PD are still unclear. Emotion perception difficulties in PD are only partially accounted for by mood disorders, cognitive decline, disease severity, and dopamine replacement therapy (Argaud et al., 2018; Coundouris et al., 2019). Theories of *emotional embodiment* suggest that individuals decode facial expressions by partially simulating what they perceive via their own musculature, thus creating motoric re-experiencing of the relevant emotions in the self (Niedenthal, 2007). Such re-experiencing, known as sensorimotor simulation, was supported by several studies (Niedenthal, 2007; Winkielman et al., 2008; Wood et al., 2016). It was suggested that hypomimia (reduction in facial expression activity in PD) disturbs facial simulation feedback, a proposed process for recognizing emotional expressions according to theories of emotional embodiment (Gray and Tickle-Degnen, 2010; Péron et al., 2014; Argaud et al., 2018).

### 1.3 Considering context: importance of body expressions in emotion perception

Prior research on emotion perception difficulties in PD largely relied on tasks using decontextualized facial expressions—that is, participants view faces in isolation. While methodologically convenient and widely popular in the neuroscience literature, this experimental approach fails to consider the fact that during most physical interactions, facial expressions are typically encountered in a rich context, not as isolated cues. Faces are perceived within a broader visual context, first and foremost the expressive body, which may be congruent or incongruent with the affective signal of the face (Abramson et al., 2017). However, the process of face-context integration in individuals with PD is poorly understood despite its potential importance (Aviezer et al., 2008, 2017). While several visual contextual factors may influence facial expression perception, body expressions constitute an immediate, intra-target source of relevant information (Wieser and Brosch, 2012). In healthy young adults, emotionally congruent body expressions appearing with the face may boost facial expression perception, while emotionally incongruent bodies may dramatically alter facial expression perception (Meeren et al., 2005; Aviezer et al., 2008, 2012; Israelashvili et al., 2019). This effect is further strengthened in the healthy aging brain, such that body expressions play a considerably larger role for older adults when perceiving face-body composites (Abo Foul et al., 2018). Specifically, healthy older adults are more influenced by incongruent body cues than their younger counterparts, emphasizing the contextual importance of bodies and how they are perceptually integrated with faces during emotion perception (Noh and Isaacowitz, 2013; Abo Foul et al., 2018).

### 1.4 Gap in research and rationale for investigation

To the best of our knowledge, the perception of composite face-body expressions in PD has not been previously investigated. This approach may be more revealing than investigating isolated cues for several reasons. First, the ecological validity of this approach bears a closer approximation to real-life social and emotion perception difficulties experienced in individuals with PD (Gunnery et al., 2016). Second, and more specifically for PD, understanding the effect of body expressions and their integrated perception of faces is essential in disorders that principally affect the body. PD produces motor symptoms in the body and face (e.g., bradykinesia and hypomimia, respectively), which can be considered a naturally occurring motor manipulation, allowing one to test the impact of impaired motor simulation feedback on emotion perception processes. Investigating the impact of motor simulation feedback deficits and their contribution to contextualized emotion perception may shed light on the underlying mechanisms of emotion perception in PD. Thus, as accumulating findings suggest different perception processing of emotions for integrated faces with bodies, investigating emotion perception from combined cues in individuals with PD is important, both clinically and theoretically. As previously emphasized, PD is a multifaceted disorder that impacts various systems, including motor, cognitive, and emotional functions, posing a challenge to attributing any singular deficit to a specific underlying mechanism. While comparing findings to healthy control groups is essential, it may not provide a comprehensive understanding. To address this complexity, we included an additional clinical control group comprising individuals with schizophrenia (SZ), a disorder that shares several intriguing parallels with PD.

### 1.5 Comparison with schizophrenia

SZ is a neuropsychiatric disorder characterized by a cluster of positive symptoms, such as hallucinations, delusions, and disorganization, as well as a cluster of negative symptoms, which include “affective flattening” (reduced emotional expressions), difficulties in emotional perception, impoverished speech, motivation, anhedonia, and decreased social interaction (for meta-analyses, see Chan et al., 2010; Kohler et al., 2010). While antipsychotic medications effectively manage positive symptoms, they can lead to Parkinsonian-like motor disturbances, encompassing bradykinesia, rigidity, and hypokinesia in facial and bodily expressions (Andreasen and Flaum, 1991; Słowiński et al., 2017; de la Mora et al., 2020). Accumulating research indicates that disruptions in dopamine neurotransmission along neural pathways play a role in the foundation of SZ (de la Mora et al., 2020; Sonnenschein et al., 2020). Individuals with SZ encounter a wide array of challenges related to emotion perception, including difficulties in recognizing emotional vocalizations (for a meta-analysis, see Gong et al., 2021), in addition to the well-documented disturbances in perceiving facial emotional expressions (for a meta-analysis, see Kohler et al., 2010), which may manifest in the early stages of the disorder (Kring and Elis, 2013). Importantly and similar to PD, individuals with SZ exhibit reduced abilities in conveying emotional facial expressions (for a review, see Trémeau, 2022).

### 1.6 Similarities and parallels between PD and SZ

PD and SZ can be viewed as two related dopamine disorders, marked by impaired and imbalanced dopamine neurotransmission (Meder et al., 2019; de la Mora et al., 2020). The presence of Parkinsonian-like movement disorders in SZ and schizophrenia-like psychosis in PD may suggest dysfunction within a shared network that encompasses the cortex, basal ganglia, thalamus, and cerebellum, while those are linked to emotion perception abilities (Pierce and Péron, 2020; Walther et al., 2020). Individuals with PD and SZ showed emotion perception difficulties, but the precise mechanism underlying these difficulties remains unclear. A comparison of PD and SZ, in addition to healthy controls, may therefore offer insights into specific and unique aspects of the emotion perception process from integrated cues in PD.

### 1.7 The current investigation

This study examined emotion perception in individuals with Parkinson's disease (PD) using integrated facial and bodily expressions. The performance of the PD group was compared to that of the HC group, as well as that of a group of individuals with SZ. The advantage of the incongruent cue approach is that the responses to the stimuli directly highlight what source (face or body) was prioritized by the perceiver in their judgment. We adopted a more ecological approach in which participants are requested to categorize the emotion of the person while being free to base their judgment on any cue they deem relevant. In addition, we examined emotion perception from isolated cues as well as from congruent faces with bodies. This allowed us to establish a baseline for the recognition of each expressive cue alone and of congruent cues in conjunction. The two clinical groups were age-matched and evaluated not only for emotion perception but also for their motor symptoms using the Unified Parkinson's Disease Rating Scale (UPDRS; Fahn and Elton, 1987). Finally, we included tests of cognition, depression, and motor function to examine if emotion perception abilities are mediated by these domains.

## 2 Method

### 2.1 Participants

Individuals with PD (*N* = 37), individuals with SZ (*N* = 30), and matched healthy controls (HC, *N* = 50) comprised our study population (see Sample Characteristics, Table 1). An *a priori* power analysis was conducted using G^*^Power3 (Faul et al., 2007) for sample size estimation, using the effect size from a related study (Abo Foul et al., 2018). Considering an alpha of 0.05 and a power of 0.95, the projected sample size needed with this effect size is approximately 36 for the main hypothesis test. The inclusion criteria for PD were (1) idiopathic PD diagnosed by a neurologist, (2) the absence of active or untreated additional neuropsychiatric disorder or psychosis, and (3) Mini Mental State Examination (MMSE) scores above 25. The inclusion criteria for SZ were (1) psychiatric diagnoses according to the criteria of the *Diagnostic and Statistical Manual of Mental Disorders, 5th edition* (DSM-5; American Psychiatric Association, 2013), (2) the absence of active or untreated neurological conditions, and (3) MMSE scores above 25. For the HC group, the inclusion criteria were (1) the absence of active neurological or neuropsychiatric conditions and (2) MMSE scores above 25.

**Table 1:** Sample characteristics and the screening tests (ACE, MMSE, FAB, MOCA, and BDI) by groups.

	**PD (*n* = 37)**	**SZ (*n* = 30)**	**HC (*n* = 49)**
Age range	28–71	30–80	27–74
Mean age (±SD)	61.41 (8.8)	57.17 (19.29)	60.80 (8.70)
Gender: female (%)	11 (30%)	13 (43%)	13 (27%)
Education: average years (±SD)	14.49 (2.91)	13.20 (2.93)	15.96 (2.06)
ACE scores: average (±SD)	89.73 (3.49) (*n* = 37)	77.62 (12.80) (*n* = 29)	94.41 (3.53) (*n* = 49)
ACE range	80–95	56–96	88–99
MMSE (±SD)	28.65 (1.11)	27.21 (2.34)	28.61 (0.98)
FAB scores: average (±SD)	15.89 (1.93) (*n* = 37)	13.40 (3.78) (*n* = 27)	16.37 (1.35) (*n* = 49)
MOCA scores: average (±SD)	25.41 (2.19) (*n* = 27)	23.23 (4.45) (*n* = 30)	26.18 (2.18) (*n* = 49)
BDI average (±SD)	11.67 (7.36) (*n* = 36)	21.28 (16.53) (*n* = 29)	(4.26) (*n* = 48)

ACE, Addenbrooke's Cognitive Examination; MMSE, Mini-Mental State Examination; FAB, Frontal Assessment Battery; MOCA, Montreal Cognitive Assessment; BDI, Beck Depression Inventory.

**Table 2:** Summary of mixed analysis of variance (ANOVA) for age, years of education, and screening tests (ACE, MMSE, FAB, MOCA, and BDI).

	***Df***	***F***	***P***
Age	(2, 113)	1.13	0.33
Education years	(2, 113)	10.90	< 0.001
MOCA	(2, 103)	9.13	< 0.001
ACE	(2, 112)	51.72	< 0.001
MMSE	(2, 112)	10.11	< 0.001
FAB	(2, 113)	15.60	< 0.001
BDI	(2, 110)	25.42	< 0.001

ACE, Addenbrooke's Cognitive Examination; MMSE, Mini-Mental State Examination; FAB, Frontal Assessment Battery; MOCA, Montreal Cognitive Assessment; BDI, Beck Depression Inventory.

Individuals with PD were recruited from the Hadassah Medical Center's neuropsychiatric clinic in Jerusalem. These participants received neuropsychological assessment as candidates for deep brain stimulation (DBS) surgery. Individuals with SZ were recruited from the Kfar Shaul Mental Health Center, Jerusalem. Characteristics of the clinical groups are shown in Table 3. Older adults were recruited either via (a) social media advertisements or (b) an online Israeli participants' pool (https://www.panel4all.co.il/pages/home.html).

**Table 3:** Clinical characteristics of Individuals with PD and SZ.

	**PD**		**SZ**		
	***n***	**Mean (SD)**	***n***	**Mean (SD)**	***p-*** **value**
Mean disease duration	37	11.62 (8.76)	27	27.19 (15.52)	< 0.001
Dominance (right)	31 (83.78%)	27 (90%)			
LEDD (mg/day)	37	1,215.9 (565.1)			
UPDRS—mentation, behavior, and mood	26	3.23 (1.50)	30	4.20 (3.16)	0.16
UPDRS—ADL	26	10.88 (5.27)	30	4.70 (4.17)	< 0.001
UPDRS—“On” motor examination	35	19.09 (8.67)	30	20.10 (9.71)	0.66
UPDRS—“On” face	26	1.00 (0.57)	30	0.93 (0.64)	0.68
UPDRS—“On” total score	26	35.65 (12.87)	30	28.47 (14.21)	0.054
UPDRS—“Off” motor examination	35	40.31 (11.66)			
UPDRS—“Off” total score	27	62.41 (16.47)			
Native language (Hebrew)	26 (70.27%)	29 (96.67%)			
First motor symptom (for PD individuals)				
Right bodyside	15				
Left bodyside	17				
Slowness	3				
Walking disturbances	2				

PD, Parkinson's disease; SZ, individuals with schizophrenia; LEDD, levodopa equivalent daily dose; UPDRS, Unified Parkinson's Disease Rating Scale.

Only one HC participant was excluded due to uncompleted experiment tasks. All those taking part had normal or corrected-to-normal vision. The study was approved by the Hebrew University of Jerusalem Ethics Committee and the IRB Committees of the Hadassah Medical Organization and Kfar Shaul Mental Health Center.

### 2.2 Stimuli: emotional expressions

**Figure 1:**
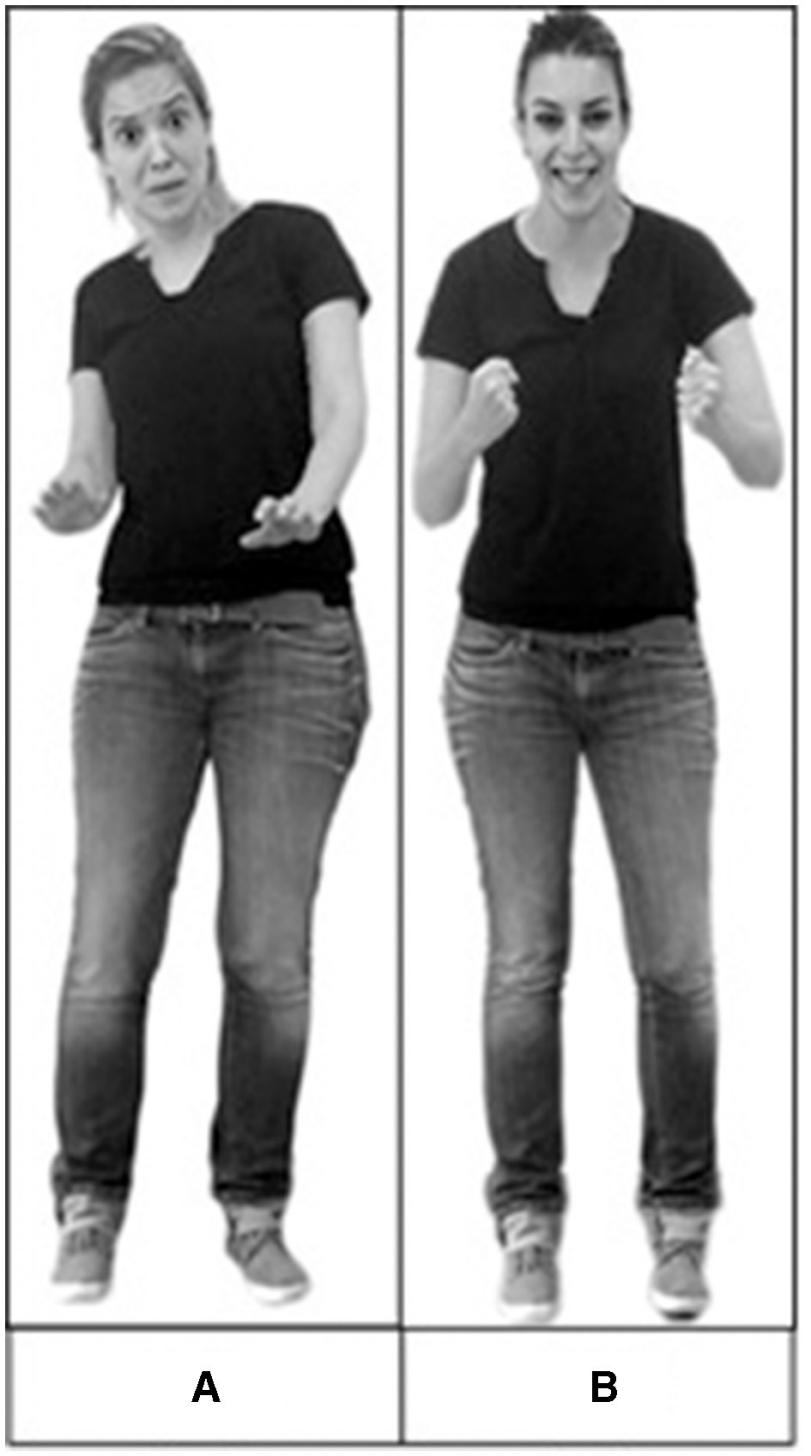
Examples of composite stimuli used in the experiment. **(A)** An example of a congruent stimulus—a fearful face combined with a fearful body. **(B)** An example of an incongruent stimulus—a happy face combined with an angry body. Reproduced with permission from the Bochum Emotional Stimulus Set (BESST) (Thoma et al., 2013) and Amsterdam Dynamic Facial Expression Set (ADFES) (van der Schalk et al., 2011).

Face-body composites were constructed with stimuli obtained from standardized sets. Stereotypical images of facial expressions of sadness, anger, fear, and happiness were taken from the Amsterdam Dynamic Facial Expression Set (ADFES) (van der Schalk et al., 2011). Stereotypical body expressions of sadness, anger, fear, and happiness were taken from the Bochum Emotional Stimulus Set (BESST) (Thoma et al., 2013). Six exemplars were used for each emotion, with equal representation across genders. Using Adobe Photoshop, we created realistically proportioned face-body composites by crossing all emotional categories of faces with bodies (Figure 1), resulting in combinations that were congruent (e.g., an angry face on an angry body) and incongruent (for example, an angry face on a fearful body). The experiment comprised three blocks, namely, (1) *body only*, (2) *face only*, and (3) *face with body*. The body-only and face-only blocks each contained 24 stimuli—six exemplars for each emotion (sadness, anger, fear, and happiness) from the sets indicated. The face-only block included isolated faces without bodies. The body-only block included bodies with blurred faces. The face-with-body block included 32 composite stimuli: eight congruent and 24 incongruent. These stimuli have been recently validated in a normal population (Lecker et al., 2017) and in healthy older adults (Abo Foul et al., 2018).

### 2.3 Cognitive assessment

#### 2.3.1 Addenbrookes' cognitive examination

ACE (Mioshi et al., 2006) is a paper-based test used for cognitive screening, characterized by good specificity and sensitivity for diagnosing different types of dementia (Mioshi et al., 2006). This test expands the Mini-Mental State Examination (MMSE) for diagnosing dementia. It examines six cognitive domains: orientation (10 points), attention (8 points), memory (35 points), verbal fluency (14 points), language (28 points), and visuospatial abilities (5 points), with a maximal score of 100. Validated versions in Hebrew (Newman, 2005), Arabic (Al Salman, 2013), and English (Mioshi et al., 2006) were used in the study, according to the native language of the participants. The internal consistency of ACE is high (Cronbach's coefficient α = 0.87), and its test-retest reliability as evaluated by the intraclass correlation coefficient (ICC) ranges from 0.64 to 0.82 (Takenoshita et al., 2019).

#### 2.3.2 Frontal assessment battery

FAB (Dubois et al., 2000) is a neuropsychological test designed to assess executive function and address dysexecutive syndrome. This 10-min test contains six domains, namely, conceptualization, mental flexibility, motor programming, sensitivity to interference, inhibitory control, and environmental autonomy. The maximum score is 18, with lower scores indicating greater impairments. The internal consistency of FAB as measured by Cronbach's coefficient α is 0.61 (Goh et al., 2019).

#### 2.3.3 Montreal Cognitive Test

MOCA (Lifshitz et al., 2012) is a widely used 10-min cognitive screening test that comprises eight cognitive domains, namely, visuospatial perception, organizational skills, recognition, naming, short-term memory, attention, verbal ability, abstraction, and orientation. In this study, we used the Hebrew, Arabic, and English versions as they appear on the MoCA test website (www.mocatest.org). The internal consistency of MoCA is high (Cronbach's coefficient α = 0.89), and its test-retest reliability as evaluated by ICC ranges from 0.64 to 0.82 (Bruijnen et al., 2020; Sala et al., 2020).

#### 2.3.4 Beck depression inventory

BDI (Beck et al., 1996) is a widely used self-report questionnaire that measures the severity of depressive symptoms. We used validated versions in Hebrew (Gil and Gilbar, 2001), English (Beck et al., 1996), and Arabic (Abdel-Khalek, 1998), tailored to the native language of the participants. The questionnaire addresses different depression symptoms, scored on a scale of 0–3 [e.g., “(0) I don't feel sad” to “(3) I'm so sad or unhappy that I can't stand it”]. Higher total scores indicate more severe depressive symptoms. The internal consistency of BDI is high (Cronbach's coefficient α = 0.9), and its test-retest reliability as evaluated by ICC ranges from 0.73 to 0.96 (Wang and Gorenstein, 2013).

### 2.4 Procedure

After providing consent, participants were assessed for cognitive state, followed by the emotion perception tests. The PD and SZ groups were assessed in person, and HC was tested online via Zoom to reduce the COVID-19 infection risk. A neurologist assessed individuals with PD and SZ for motor symptoms using the UPDRS (Fahn and Elton, 1987). At the time of testing, the PD and SZ groups were taking their regular dopaminergic and antipsychotic medications, respectively. Following cognitive assessment, participants performed the experimental emotion perception tasks. Patients were tested in person using an E-Prime-controlled computer, while healthy controls were tested using *Gorilla* (Anwyl-Irvine et al., 2019), an online experiment platform. Blocks (face, body, and face with body) and stimuli within each block were randomly ordered. Each stimulus appeared for 2,000 ms, but no time limit was imposed for responses. Participants were instructed to select the emotion that best reflected the target's feelings from a list of four labels, namely, fear, anger, sadness, and happiness. In the case of face-body composites, no instructions were given prioritizing the face or body; rather, responses were to be made based on the overall impression of the target's emotion. BDI was completed after the session.

### 2.5 Analysis strategy

#### 2.5.1 Perception of emotion from incongruent composites

Because participants were not requested to base their answer on the face or the body, incongruent face–body composites do not yield an objective “accurate” response. Therefore, to quantify the recognition of emotion from incongruent composites, we analyzed the tendency to categorize the composites as conveying the emotion of the face, the body, or neither. This was done using a 3 (groups: PD, HC, and SZ) × 3 (categorization tendencies: as face, as body, and other) mixed ANOVA. As recent meta-analyses do not support substantial differences as a function of specific emotions (Coundouris et al., 2019), we pooled the main results across emotions. A detailed exploratory analysis of group differences broken down to specific facial emotional expressions is also provided in the Supplementary material.

#### 2.5.2 Perception of faces, bodies, and congruent face-body composites

To assess affective perception, we calculated the mean perception accuracy of each participant separately for each emotion in each cue. As no specific *a priori* predictions were raised for differences in the perception of specific emotions, accuracy rates were pooled across emotions. The mean perception accuracy of each participant pooled across emotions was subjected to a 3 (groups: PD, HC, and SZ) × 3 (cues: isolated face, isolated body, and congruent faces with bodies) ANOVA. A detailed exploratory analysis with specific emotions is also provided in the Supplementary material using a 3 (groups: PD, HC, and SZ) × 3 (cues: isolated face, isolated body, and congruent face-body) × 4 (emotions: anger, sadness, fear, and happiness) mixed ANOVA.

#### 2.5.3 Additional analysis

We also examined the correlations between affective perception accuracy, categorization tendency, cognitive screening test scores, BDI, years of education, and motor UPDRS scores. When required, *p*-values were adjusted using Greenhouse-Geisser correction, with Bonferroni corrections applied for follow-up *t*-tests.

## 3 Results

### 3.1 Demographic and clinical characteristics

The demographic, cognitive, and clinical characteristics of the experiment groups are shown in Tables 1–3. The groups were age-matched (*p* = 0.33) and, importantly, no differences were found between the clinical groups in the UPDRS motor score (*p* = 0.66). Predictably, the groups differed in cognitive performance: HC scored highest in ACE, followed by individuals with PD, while individuals with SZ scored lowest (all *t*-contrasts *p* < 0.001). The PD and HC groups showed comparable cognitive performance in MMSE and FAB (both *p* = 1), whereas the SZ group was the lowest (all *t*-contrasts *p* < 0.001). Individuals with SZ scored highest for depression in the BDI, followed by those with PD, with HC reporting the lowest scores (all *t*-contrasts *p* < 0.001). The PD and SZ groups had comparable levels of education (*p* = 0.14), which were lower than those in the HC group (all *t*-contrasts *p* < 0.01).

### 3.2 Perception of emotionally incongruent faces with bodies in PD, SZ, and HC

**Figure 2:**
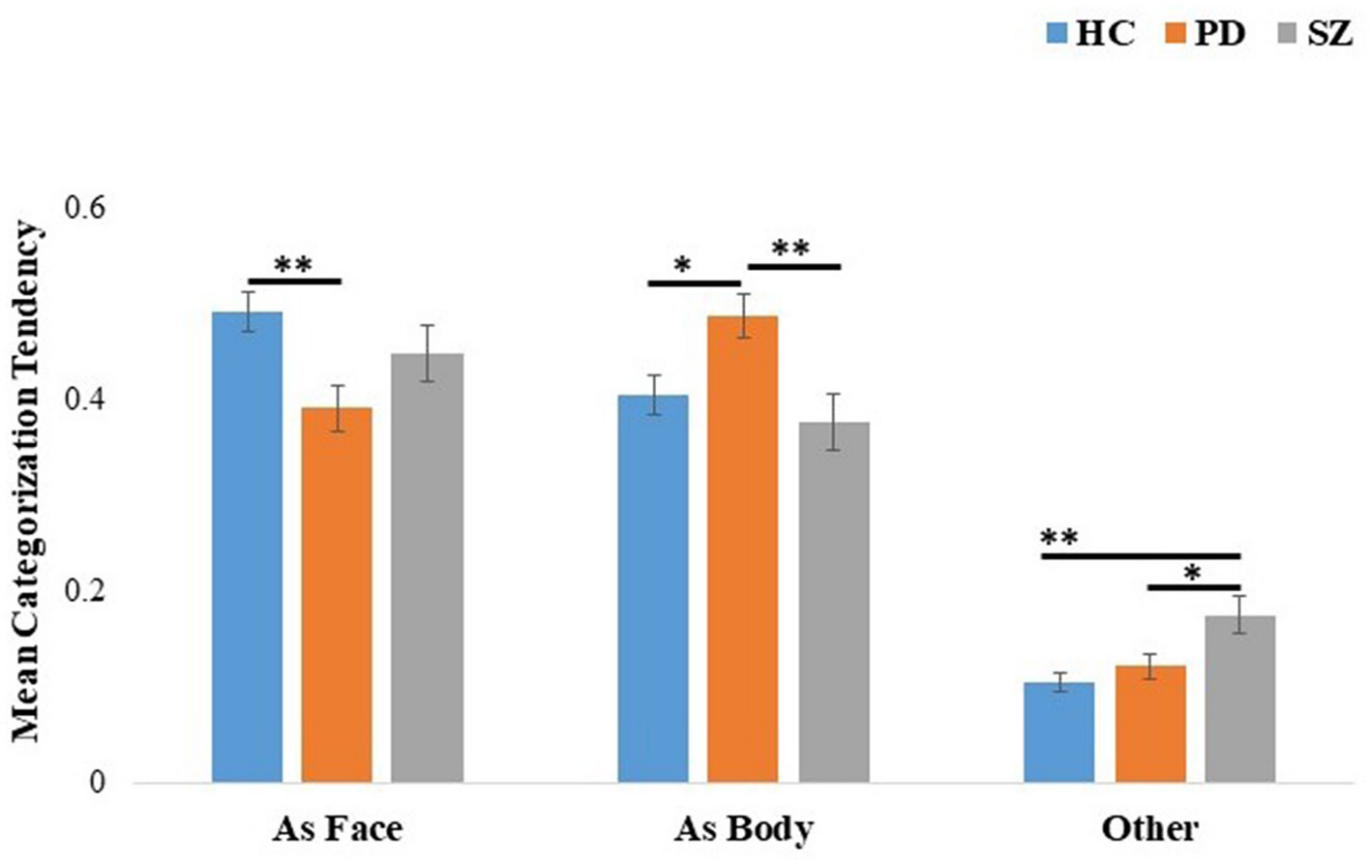
Mean categorization tendency of incongruent face-body composite stimuli for the HC, PD, and SZ groups. The categorization tendency [as face-emotion, body-emotion, and other (categorizations do not correspond to face or body emotions)] is shown on the *x*-axis. Error bars represent standard errors. *p < 0.05, **p < 0.01. HC, healthy controls; PD, Parkinson's disease group; SZ, schizophrenia group.

A 3 (groups: PD, SZ, and HC) × 3 (categorization tendencies: as face, as body, and as other) mixed ANOVA was performed on the categorizations (see Figure 2). Significant effects were found for categorization tendency, *F*_(2, 226)_ = 139.35, *p* < 0.001, η^2^_*p*_ = 0.55, and for the interaction of group × categorization tendency, *F*_(2, 226)_ = 5.64, *p* < 0.001, η^2^_*p*_ = 0.09. Follow-up comparisons revealed that individuals with PD were more likely to be affected by body cues than the HC (*p* = 0.026) and SZ groups (*p* = 0.006). HC had a greater tendency to categorize the composites according to facial expression than the PD group (*p* = 0.006) but no difference was found between the HC and SZ groups (*p* = 0.64). Individuals with SZ were more likely than HC (*p* = 0.001) and more likely than individuals with PD (*p* = 0.03) to categorize composites as conveying an emotion not presented in the face or body.

### 3.3 Group differences in emotion perception: isolated faces, isolated bodies, and emotionally congruent faces with bodies

**Figure 3:**
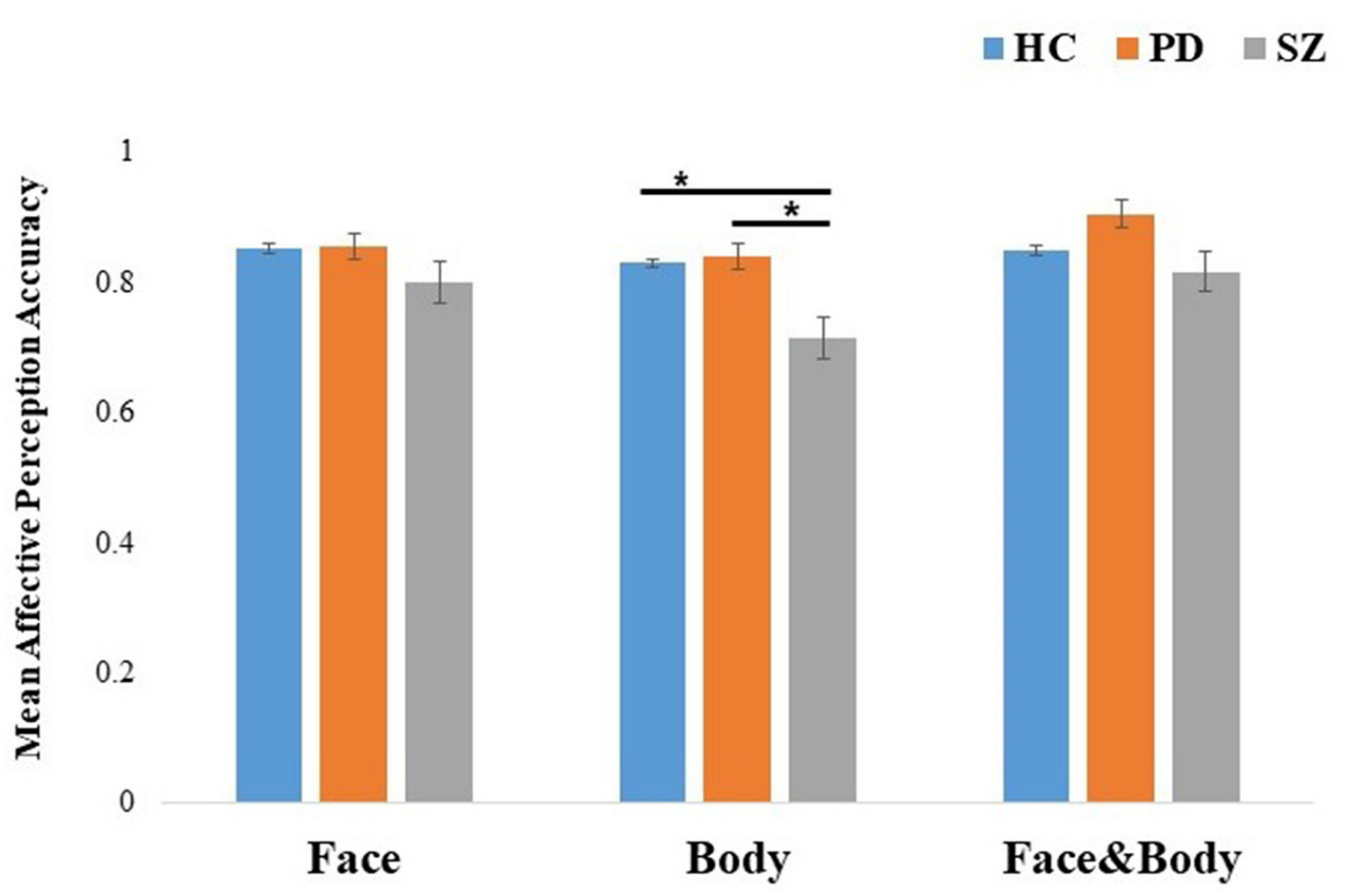
Mean effective perception accuracy of emotion recognition by cue: face, body, and congruent faces with bodies for HC, PD, and SZ. The results were pooled across emotions. Error bars represent standard errors. *p < 0.001. HC, healthy controls; PD, Parkinson's disease group; SZ, schizophrenia group.

A 3 (groups: PD, SZ, and HC) × 3 (cues: face, body, and face with body) mixed ANOVA was run on the mean affective perception accuracy per cue for each participant (see Figure 3). The results revealed significant effects for cue, *F*_(2, 226)_ = 12.05, *p* < 0.001, η^2^_*p*_ = 0.10, group, *F*_(2, 113)_ = 6.56, *p* = 0.002, η^2^_*p*_ = 0.10, and importantly for the cue × group interaction, *F*_(4, 226)_ = 2.54, *p* = 0.04, η^2^_*p*_ = 0.04. To interpret the interaction effects, comparisons between the experimental groups for each cue were performed. For isolated faces, the groups showed comparable mean affective perception accuracy (*p* = 0.07). For isolated bodies, the SZ group showed significantly worse recognition compared to both the PD and HC groups (both *ps* = 0.001), while the PD group did not differ from the HC group (*p* = 0.9). Finally, a trend was found in differences for the perception of emotionally congruent faces with bodies across groups (*p* = 0.049).

To strengthen our inferences concerning the lack of difference in comparing PD and HC groups in the perception of isolated cues, we conducted Bayesian comparisons. The corresponding Bayes factors suggested moderate evidence for the null hypothesis relative to the alternative hypothesis in the affective perception of isolated faces: BF_01_ = 4.38 and for isolated bodies: BF_01_ = 4.144.

### 3.4 Correlations between the categorization tendency of incongruent composites with cognitive screening tests, BDI, motor UPDRS, and education years

We next examined whether the categorization tendency in emotionally incongruent faces and bodies is related to participant performance in the cognitive tests, depression scores from the BDI questionnaire, motor state from the UPDRS score, and educational level by calculating Pearson's bivariate correlations. Full results are shown in Supplementary Table S2. No significant correlations were found in any of the groups between the tendency to categorize incongruent composites as face or body and participant performance in the cognitive tests (ACE, MoCA, and FAB), their averaged years of education, or their BDI scores. No significant correlations were found between the tendency to categorize as body or face and the motor UPDRS scores and UPDRS face scores of the clinical groups (*p* > 0.1).

### 3.5 Correlations between the mean affective perception accuracy of isolated faces, isolated bodies, and congruent faces with bodies with cognitive screening tests, BDI, motor UPDRS, and education years

To examine whether the mean affective perception of the isolated faces, isolated bodies, or congruent faces and bodies is related to participants performance in cognitive screening, depression, motor UPDRS, and educational level, Pearson's bivariate correlations were calculated, see Supplementary Table S3 for full results. Results show that in the SZ group, higher cognitive performance, as tested by MOCA and ACE, was positively correlated with better emotion perception of the isolated body (*r* = 0.43 and *r* = 0.43, respectively) and isolated facial expressions (*r* = 0.55 and *r* = 0.57, respectively). Significant correlations were found between FAB scores and recognition of isolated facial (*r* = 0.50), body (*r* = 0.43), and congruent faces with bodies (*r* = 0.42) in the SZ group, indicating that higher scores in executive functions are positively correlated with the perception of all of the emotional cues. Additionally, a significant correlation was found between the UPDRS motor score and the mean perception of facial expressions (*r* = −0.43), but not with isolated bodily expressions or congruent faces with bodies.

In the PD group, significant positive correlations were found between the mean affective perception accuracy of isolated bodies and MOCA, *r* = 0.41, and FAB scores, *r* = 0.44. In the HC group, there were significant positive correlations between the mean affective perception accuracy of isolated bodies and MOCA scores, *r* = 0.29, and the mean affective perception accuracy of isolated faces and FAB scores, *r* = 0.38. Additionally, despite the lack of statistically significant findings, there were weak negative correlations (ranging from −0.25 to −0.31) observed between UPDRS facial scores and the average accuracy of emotional cue perception.

While not reaching statistical significance, it is noteworthy that a correlation emerged between ACE scores and the mean perception accuracy of emotional cues in both the HC and PD groups (*r* ranged from 0.23 to 0.31), suggesting a moderate effect size. Together with SZ group findings, the results suggested that individuals who performed worse at selecting the correct emotion in the face or body had lower cognition scores.

## 4 Discussion

The objective of this study was to examine emotion perception among individuals with PD by assessing their ability to integrate emotionally incongruent face-body composites as well as these cues presented individually and congruently. PD performance was compared to HC and, more importantly, to the SZ group. The results demonstrated that when incongruent face-body expressions were presented as composites, individuals with PD tended to categorize them based on the body's emotion, while HC predominantly categorized them according to the facial emotion. Notably, there was no consistent inclination toward prioritizing either the face or the body in the case of individuals with SZ. When faces and bodies were perceived in isolation, no significant differences emerged between PD and HC in our sample. In contrast, the SZ group showed significantly worse recognition of isolated bodies compared to both the PD and HC groups. When emotionally congruent faces with bodies were presented together, no significant differences were found between the research groups. Thus, the differences between individuals with PD, SZ, and HCs in prioritizing the body over the face could not be explained by the better accuracy or clarity of the isolated cues that formed the composite stimuli. Furthermore, no substantial correlations were identified between cognitive abilities, executive functions, and the categorization tendencies for incongruent composites observed in individuals with PD, SZ, and HC.

### 4.1 Exploring emotion perception abilities in PD and SZ

The reason for the comparable recognition of isolated faces in individuals with Parkinson's disease (PD) and healthy controls (HC) in our study remains unclear. While such findings are not rare in the literature—approximately one-third of studies indicate no differences in the perception of isolated facial expressions in PD—the typical results demonstrate poorer emotion recognition in PD compared to controls. One potential explanation could be linked to the specific characteristics of the PD individuals preselected as suitable candidates for Deep Brain Stimulation (DBS) procedures. According to DBS referral guidelines, these individuals have idiopathic PD without dementia and lack uncontrolled additional neuropsychiatric disorders (Silberstein et al., 2009). This unique subgroup of PD individuals might contribute to the observed comparable recognition of isolated facial expressions, suggesting that individuals selected for DBS may have a distinct profile or progression of PD impacting facial expression recognition. A second plausible explanation involves dopaminergic replacement therapy (DRT). DRT could influence the perception of facial expressions in two ways: (1) in the late stages of the disease, it may have a beneficial effect (Péron et al., 2014). (2) DRT activates the default mode network, enhancing attentional resources to external cues and emotion perception consequently (Delaveau et al., 2010). In the current investigation, individuals with PD were in the late stages of the disease and were examined while on their regular DRT.

Our results are in accordance with previous findings showing decreased emotion perception abilities in SZ. Individuals with SZ frequently face difficulties in accurately identifying and understanding emotional cues, leading to compromised social functioning and interpersonal difficulties. Kohler et al. (2010) meta-analysis revealed consistent impairments in facial emotion recognition among individuals with SZ, indicating a specific vulnerability in processing facial expressions. Green et al. (2015) further associated emotion perception deficits with poorer functional outcomes in schizophrenia, underscoring the clinical relevance of these impairments.

### 4.2 Exploring contextualized emotion perception in PD and SZ: insights from facial and body cues integration

Emotion perception abilities were widely investigated in various neuropsychiatric conditions, including SZ and PD, while those usually based on emotion perception of facial expressions were void of context. Recent studies stress the critical role of emotional context—specifically, emotional body context—in perceiving emotion. Information derived from body expression is utilized to disambiguate facial expressions (Aviezer et al., 2008, 2012, 2017). In healthy aging, older adults who perceive incongruent cues are more affected by body vs. face cues than young adults (Abo Foul et al., 2018). In this sense, a response pattern prioritizing body context over faces may reflect an adaptive compensatory approach, potentially beneficial in real-life conditions when a face may be more ambiguous than its context (Abramson et al., 2017; Israelashvili et al., 2019).

It is noteworthy that only a limited number of studies have explored PD individual's capacity to integrate information from multiple cues, whether unimodal or multimodal. For example, Fearon et al. (2015) demonstrated multi-modal facilitation in PD patients using congruent audiovisual stimuli. In our study, participants perceiving composite stimuli were not instructed to base their responses on the face or body but were encouraged to form an overall impression of the expressive target, mirroring daily social interactions. Furthermore, our sample of individuals with PD exhibited proficient perception of both facial and bodily cues when presented independently. These findings raise intriguing questions about the nature and implications of contextualized emotional expression perception in PD and its intricate interplay with other aspects of the disorder.

In the context of receiving multiple emotional cues, individuals with SZ face challenges in multi-sensory integration, as highlighted by Lin et al. (2020) review, suggesting difficulties at different stages of emotional processing. Reflecting on our results, individuals with SZ in the current study were worse at recognizing body emotions and exhibited no consistent pattern in integrating incongruent facial and bodily cues. Furthermore, when they showed incongruent face-body composites, they displayed a disproportionate level of responses that did not fit the face or the body. This response pattern was absent in SZ, suggesting emotion perception deficits may contribute to their social interaction difficulties. Altogether, these findings suggest a breakdown in emotion perception capabilities, especially when the target is complex and potentially ambiguous.

The distinct emotional perception patterns in PD and SZ, despite shared motor symptoms and affective flattening, may hint that motor symptomology alone cannot explain the observed differences. In our study, emotional perception patterns concerning incongruent cues did not correlate with the severity of motor symptoms in the face or the body in either the PD or SZ groups. In line with recent work (Vannuscorps et al., 2020), the current results challenge the hypothesized link between mimicry and emotion perception (Ricciardi et al., 2015, 2017), with embodiment theories positing that recognizing emotional states in others necessitates the simulation of the motor production of the perceived emotional expression, known as sensorimotor simulation (Wood et al., 2016).

In a comprehensive review of the integration of cues from the whole person, Hu et al. (2020) suggest two centers of face-body integration, namely, (1) the dorsal social agent hub that integrates face and body and other temporally synchronous cues and (2) the ventral semantic visual hub, which assimilates contextually semantic information. Although this model does not explicitly address emotion processing in the whole person, our findings, derived from comparisons between neuropsychiatric groups, may present preliminary evidence for the separate processing of emotion perception based on integrated facial and body cues. Exploring the utilization of whole-person cues could prove to be a valuable focus for future research, offering a naturalistic approach that contributes both theoretical and clinical insights to the study of emotion perception.

### 4.3 Limitations and future directions

Several limitations should be noted in the current work. First, although it included combined facial and body expressions, which are closer to what one encounters in real-life interactions, the stimuli were posed, stereotypical, and static emotional expressions. Recent findings stress the importance of investigating emotional perception using dynamic stimuli, especially in older adults (Abo Foul et al., 2022). Future research utilizing more ecologically oriented stimuli is needed. Second, despite attempts to ensure the matching of age among the experimental groups and of motor UPDRS scores among the clinical groups, differences in cognitive performance were noted. While these differences could potentially influence emotional perception abilities, we believe they are unlikely to account for our primary findings, as correlations with the cognitive scores were found in all of the experiment groups. Future research with larger samples is needed to explore the effects of cognitive abilities and other potential moderators on face-body emotion perception, such as gender. Third, the study focused exclusively on a single aspect of social perception—the perception of emotional visual stimuli. Future studies should broaden their scope to include a wider range of social perception aspects (Castro and Isaacowitz, 2019; Schlegel et al., 2020). Fourth, healthy controls were assessed online, whereas the clinical groups were evaluated in person. Although it remains unclear how such differences might contribute to the specific pattern of results observed, it is plausible that differences in testing methods might yield overall broader discrepancies in results. Consequently, further research conducted under similar experimental conditions is warranted.

Finally, additional exploration is warranted to better understand the relationship between facial motor symptoms and emotion perception. The present investigation may lack sensitivity in revealing connections between facial masking and emotion perception abilities for two main reasons. First, the sample size may be insufficiently powered to detect subtle differences, and second, the UPDRS assessment of facial motor symptoms is somewhat rudimentary and subjective. Future research examining the link between motor symptomatology and emotion perception could benefit from incorporating additional measures, such as electromyography (EMG) and automated video analysis, to assess muscle activity in facial expressions while also employing a larger sample size. This more comprehensive approach would provide valuable insights into the intricate interplay between facial motor function and the perception of emotions.

### 4.4 Caveats and conclusion

The current study found that individuals with PD showed an increased tendency to categorize incongruent face-body combinations in line with the body emotion, whereas those with HC showed a tendency to classify them in line with the facial emotion. Importantly, individuals with SZ showed no consistent pattern alongside responses that did not correspond to the face or body. These results were not explained by the recognition of the isolated face or body cues, cognitive status, depression, or motor symptoms. As real-life expressions may include inconsistent cues in the body and face, these findings may have implications for the way individuals with PD and SZ interpret the emotions of others.

## Data availability statement

The raw data supporting the conclusions of this article will be made available by the authors, without undue reservation.

## Ethics statement

The studies involving humans were approved by the Hebrew University of Jerusalem Ethics Committee and the IRB Committees of the Hadassah Medical Organization and Kfar Shaul Mental Health Center. The studies were conducted in accordance with the local legislation and institutional requirements. The participants provided their written informed consent to participate in this study.

## Author contributions

YA: Formal analysis, Investigation, Project administration, Visualization, Writing – original draft, Writing – review & editing. DA: Writing – review & editing. AD: Writing – review & editing. YN: Writing – review & editing. EL: Writing – review & editing. MA: Writing – review & editing. HA: Conceptualization, Supervision, Writing – review & editing. RE: Conceptualization, Resources, Supervision, Writing – review & editing.

## References

[B1] Abdel-Khalek A. M. (1998). Death, anxiety, and depression in Lebanese undergraduates. OMEGA-J. Death Dying 37, 289–302. 10.2190/CN5K-XF4C-2NPG-17E022612255

[B2] Abo Foul Y. Eitan R. Aviezer H. (2018). Perceiving emotionally incongruent cues from faces and bodies: older adults get the whole picture. Psychol. Aging 33:660. 10.1037/pag0000255 29902057

[B3] Abo Foul Y. Eitan R. Mortillaro M. Aviezer H. (2022). Perceiving dynamic emotions expressed simultaneously in the face and body minimizes perceptual differences between young and older adults. J. Gerontol. Ser. B, 77, 84–93. 10.1093/geronb/gbab064 33842959

[B4] Abramson L. Marom I. Petranker R. Aviezer H. (2017). Is fear in your head? A comparison of instructed and real-life expressions of emotion in the face and body. Emotion 17, 557–565. 10.1037/emo0000252 27929305

[B5] Al Salman A. S. A. (2013). The Saudi Arabian adaptation of the Addenbrooke's Cognitive Examination–Revised (Arabic ACE-R) (Doctoral dissertation). University of Glasgow.

[B6] American Psychiatric Association (2013). Diagnostic and Statistical Manual of Mental Disorders, 5th Edn. 10.1176/appi.books.9780890425596

[B7] Andreasen N. C. Flaum M. (1991). Schizophrenia: the characteristic symptoms. Schizophr. Bull. 17, 27–49. 10.1093/schbul/17.1.27 2047788

[B8] Anwyl-Irvine A. L. Massonié J. Flitton A. Kirkham N. Z. Evershed J. K. (2019). Gorilla in our midst: an online behavioural experiment builder. Behav. Res. Methods. 52, 388–407. 10.3758/s13428-019-01237-x 31016684 PMC7005094

[B9] Argaud S. Vérin M. Sauleau P. Grandjean D. (2018). Facial emotion recognition in Parkinson's disease: a review and new hypotheses. Mov. Disord. 33, 554–567. 10.1002/mds.27305 29473661 PMC5900878

[B10] Assogna F. Pontieri F. E. Caltagirone C. Spalletta G. (2008). The recognition of facial emotion expressions in Parkinson's disease. Eur. Neuropsychopharmacol. 18, 835–848. 10.1016/j.euroneuro.2008.07.004 18707851

[B11] Aviezer H. Ensenberg N. Hassin R. R. (2017). The inherently contextualized nature of facial emotion perception. Curr. Opin. Psychol. 17, 47–54. 10.1016/j.copsyc.2017.06.006 28950972

[B12] Aviezer H. Hassin R. R. Ryan J. Grady C. Susskind J. Anderson A.. (2008). Angry, disgusted, or afraid? Studies on the malleability of emotion perception. Psychol. Sci. 19, 724–732. 10.1111/j.1467-9280.2008.02148.x 18727789

[B13] Aviezer H. Trope Y. Todorov A. (2012). Body cues, not facial expressions, discriminate between intense positive and negative emotions. Science 338, 1225–1229. 10.1126/science.1224313 23197536

[B14] Beck A. Steer R. Brown G. (1996). Beck depression inventory-II. San Antonio 78, 490–498. 10.1037/t00742-000

[B15] Bernal-Pacheco O. Limotai N. Go C. L. Fernandez H. H. (2012). Nonmotor manifestations in Parkinson disease. Neurologist 18, 1–16. 10.1097/NRL.0b013e31823d7abb 22217609

[B16] Bruijnen C. J. W. H. Dijkstra B. A. G. Walvoort S. J. W. Budy M. J. J. Beurmanjer H. De Jong C. A. J.. (2020). Psychometric properties of the Montreal Cognitive Assessment (MoCA) in healthy participants aged 18-70. Int. J. Psychiatry Clin. Pract. 24, 293–300. 10.1080/13651501.2020.1746348 32271127

[B17] Castro V. Isaacowitz D. (2019). The same with age: Evidence for age-related similarities in interpersonal accuracy. J. Exp. Psychol. 148, 1517–1537. 10.1037/xge0000540 30550339 PMC6682457

[B18] Chan R. C. Li H. Cheung E. F. Gong Q. Y. (2010). Impaired facial emotion perception in schizophrenia: a meta-analysis. Psychiatry Res. 178, 381–390. 10.1016/j.psychres.2009.03.035 20483476

[B19] Coundouris S. Adams A. Grainger S. Henry J. (2019). Social perceptual function in Parkinson's disease: a meta-analysis. Neurosci. Biobehav. Rev. 104, 255–267. 10.1016/j.neubiorev.2019.07.011 31336113

[B20] de la Mora M. P. Hernandez-Mondragon C. Crespo-Ramirez M. Rejon-Orantes J. Borroto-Escuela D. O. Fuxe K. (2020). Conventional and novel pharmacological approaches to treat dopamine-related disorders: focus on Parkinson's disease and schizophrenia. Neuroscience 439, 301–318. 10.1016/j.neuroscience.2019.07.026 31349007

[B21] Delaveau P. Salgado-Pineda P. Fossati P. Witjas T. Azulay J. P. Blin O. (2010). Dopaminergic modulation of the default mode network in Parkinson's disease. Eur. Neuropsychopharmacol. 20, 784–792. 10.1016/j.euroneuro.2010.07.001 20674286

[B22] Dubois B. Slachevsky A. Litvan I. Pillon B. (2000). The FAB: a frontal assessment battery at bedside. Neurology 55, 1621–1626. 10.1212/WNL.55.11.1621 11113214

[B23] Fahn S. Elton R. (1987). “Unified Parkinson's disease rating scale,” in Recent Developments in Parkinson's Disease, Vol 2, S. Fahn, C. D. Marsden, D. B. Calne, and M. Goldstein (Florham Park, NJ: Macmillan Health Care Information), 153–163, 293–304

[B24] Faul F. Erdfelder E. Lang A. G. Buchner A. (2007). G^*^ Power 3: a flexible statistical power analysis program for the social, behavioral, and biomedical sciences. Behav. Res. Methods 39, 175–191. 10.3758/BF03193146 17695343

[B25] Fearon C. Butler J. Newman L. Lynch T. Reilly R. (2015). Audiovisual processing is abnormal in Parkinson's disease and correlates with freezing of gait and disease duration. J. Parkinsons Dis. 5, 925–936. 10.3233/JPD-150655 26485427

[B26] Gelb D. J. Oliver E. Gilman S. (1999). Diagnostic criteria for Parkinson disease. Arch. Neurol. 56, 33–39. 10.1001/archneur.56.1.33 9923759

[B27] Gil S. Gilbar O. (2001). Hopelessness among cancer patients. J. Psychosoc. Oncol. 19, 21–33. 10.1300/J077v19n01_02

[B28] GohW. Y.ChanD.AliN. B.ChewA. P.ChuoA.ChanM.. (2019). Frontal assessment battery in early cognitive impairment: psychometric property and factor structure. J. Nutr. Health Aging 23, 966–972. 10.1007/s12603-019-1248-031781726

[B29] Gong B. Li Q. Zhao Y. Wu C. (2021). Auditory emotion recognition deficits in schizophrenia: a systematic review and meta-analysis. Asian J. Psychiatr. 65:102820. 10.1016/j.ajp.2021.102820 34482183

[B30] Gothwal M. Arumugham S. S. Yadav R. Pal P. K. Hegde S. (2022). Deficits in emotion perception and cognition in patients with Parkinson's disease: a systematic review. Ann. Indian Acad. Neurol. 25, 367–375. 10.4103/aian.aian_573_21 35936598 PMC9350746

[B31] Gray H. M. Tickle-Degnen L. (2010). A meta-analysis of performance on emotion recognition tasks in Parkinson's disease. Neuropsychology 24, 176–191. 10.1037/a0018104 20230112

[B32] Green M. F. Horan W. P. Lee J. (2015). Social cognition in schizophrenia. Nat. Rev. Neurosci. 16, 620–631. 10.1038/nrn4005 26373471

[B33] Gunnery S. D. Habermann B. Saint-Hilaire M. Thomas C. A. Tickle-Degnen L. (2016). The relationship between the experience of hypomimia and social wellbeing in people with Parkinson's disease and their care partners. J. Parkinsons Dis. 6, 625–630. 10.3233/JPD-160782 27285568 PMC5003752

[B34] Hayes G. McLennan S. Henry J. Phillips L. Terrett G. Rendell P.. (2020). Task characteristics influence facial emotion perception age-effects: a meta-analytic review. Psychol. Aging 35:295. 10.1037/pag0000441 31999152

[B35] Hindle J. Martyr A. Clare L. (2014). Cognitive reserve in Parkinson's disease: a systematic review and meta-analysis. Parkinson. Relat. Disord. 20, 1–7. 10.1016/j.parkreldis.2013.08.010 24034887

[B36] Hu Y. Baragchizadeh A. O'Toole A. J. (2020). Integrating faces and bodies: psychological and neural perspectives on whole person perception. Neurosci. Biobehav. Rev. 112, 472–486. 10.1016/j.neubiorev.2020.02.021 32088346

[B37] Israelashvili J. Hassin R. R. Aviezer H. (2019). When emotions run high: a critical role for context in the unfolding of dynamic, real-life facial affect. Emotion 19, 558–562. 10.1037/emo0000441 29985010

[B38] Jacobs D. H. Shuren J. Bowers D. Heilman K. M. (1995). Emotional facial imagery, perception, and expression in Parkinson's disease. Neurology 45, 1696–1702. 10.1212/WNL.45.9.1696 7675229

[B39] Kohler C. Walker J. Martin E. Healey K. Moberg P. (2010). Facial emotion perception in schizophrenia: a meta-analytic review. Schizophr. Bull. 36, 1009–1019. 10.1093/schbul/sbn192 19329561 PMC2930336

[B40] Kring A. M. Elis O. (2013). Emotion deficits in people with schizophrenia. Annu. Rev. Clin. Psychol. 9, 409–433. 10.1146/annurev-clinpsy-050212-185538 23245340

[B41] Lecker M. Shoval R. Aviezer H. Eitam B. (2017). Temporal integration of bodies and faces: United we stand, divided we fall? Vis. Cogn. 25, 477–491. 10.1080/13506285.2017.1310164

[B42] Lifshitz M. Dwolatzky T. Press Y. (2012). Validation of the Hebrew version of the MoCA test as a screening instrument for the early detection of mild cognitive impairment in elderly individuals. J. Geriatr. Psychiatry Neurol. 25, 155–161. 10.1177/0891988712457047 23124009

[B43] Lin C. Tien Y. Huang J. Tsai C. Hsu L. (2016). Degraded impairment of emotion recognition in Parkinson's disease extends from negative to positive emotions. Behav. Neurol. 2016:9287092. 10.1155/2016/9287092 27555668 PMC4983334

[B44] Lin Y. Ding H. Zhang Y. (2020). Multisensory integration of emotion in schizophrenic patients. Multisens. Res. 33, 865–901. 10.1163/22134808-bja10016 33706267

[B45] Meder D. Herz D. Rowe J. Lehéricy S. Siebner H. (2019). The role of dopamine in the brain-lessons learned from Parkinson's disease. Neuroimage 190, 79–93. 10.1016/j.neuroimage.2018.11.021 30465864

[B46] Meeren H. K. van Heijnsbergen C. C. de Gelder B. (2005). Rapid perceptual integration of facial expression and emotional body language. Proc. Natl. Acad. Sci. U. S. A. 102, 16518–16523. 10.1073/pnas.0507650102 16260734 PMC1283446

[B47] Mioshi E. Dawson K. Mitchell J. Arnold R. Hodges J. (2006). The Addenbrooke's Cognitive Examination Revised (ACE-R): a brief cognitive test battery for dementia screening. Int. J. Geriatr. Psychiatry 21, 1078–1085. 10.1002/gps.1610 16977673

[B48] Newman J. (2005). Brief assessment of cognitive mental status in Hebrew: Addenbrooke's Cognitive Examination. IMAJ-Ramat Gan 7:451. 16011062

[B49] Niedenthal P. M. (2007). Embodying emotion. Science 316, 1002–1005. 10.1126/science.1136930 17510358

[B50] Noh S. R. Isaacowitz D. M. (2013). Emotional faces in context: age differences in recognition accuracy and scanning patterns. Emotion 13, 238–249. 10.1037/a0030234 23163713 PMC4119600

[B51] Pell M. Leonard C. (2003). Processing emotional tone from speech in Parkinson's disease: a role for the basal ganglia. Cogn. Affect. Behav. Neurosci. 3, 275–288. 10.3758/CABN.3.4.275 15040548

[B52] Péron J. Grandjean D. Drapier S. Vérin M. (2014). Effect of dopamine therapy on nonverbal affect burst recognition in Parkinson's disease. PLoS ONE 9:e90092. 10.1371/journal.pone.0090092 24651759 PMC3961247

[B53] Phillips M. L. Drevets W. C. Rauch S. L. Lane R. (2003). Neurobiology of emotion perception I: the neural basis of normal emotion perception. Biol. Psychiatry 54, 504–514. 10.1016/S0006-3223(03)00168-9 12946879

[B54] Pierce J. E. Péron J. (2020). The basal ganglia and the cerebellum in human emotion. Soc. Cogn. Affect. Neurosci. 15, 599–613. 10.1093/scan/nsaa076 32507876 PMC7328022

[B55] Ricciardi L. Bologna M. Morgante F. Ricciardi D. Morabito B. Volpe D.. (2015). Reduced facial expressiveness in Parkinson's disease: a pure motor disorder? J. Neurol. Sci. 358, 125–130. 10.1016/j.jns.2015.08.1516 26365284

[B56] Ricciardi L. Visco-Comandini F. Erro R. Morgante F. Bologna M. Fasano A.. (2017). Facial emotion recognition and expression in Parkinson's disease: an emotional mirror mechanism? PLoS ONE 12:e0169110. 10.1371/journal.pone.0169110 28068393 PMC5221788

[B57] Sala G. Inagaki H. Ishioka Y. Masui Y. Nakagawa T. Ishizaki T.. (2020). The psychometric properties of the Montreal Cognitive Assessment (MoCA): a comprehensive investigation. Swiss J. Psychol. 79, 155–161. 10.1024/1421-0185/a000242

[B58] Schlegel K. Vicaria M. Isaacowitz D. (2020). Facets of interpersonal accuracy across the lifespan: is there a single skill in older age? J. Nonverb. Behav. 1–26. 10.1007/s10919-019-00326-x

[B59] Schneider F. Althaus A. Backes V. Dodel R. (2008). Psychiatric symptoms in Parkinson's disease. Eur. Arch. Psychiatry Clin. Neurosci. 258(Suppl. 5), 55–59. 10.1007/s00406-008-5012-4 18985296

[B60] Schröder C. Nikolova Z. Dengler R. (2010). Changes of emotional prosody in Parkinson's disease. J. Neurol. Sci. 289, 32–35. 10.1016/j.jns.2009.08.038 19732910

[B61] Silberstein P. Bittar G. Boyle R. Cook R. Coyne T. O'Sullivan D.. (2009). Deep brain stimulation for Parkinson's disease: Australian referral guidelines. J. Clin. Neurosci. 16, 1001–1008. 10.1016/j.jocn.2008.11.026 19596113

[B62] Simons G. Pasqualini M. C. Reddy V. Wood J. (2004). Emotional and nonemotional facial expressions in people with Parkinson's disease. J. Int. Neuropsychol. Soc. 10, 521–535. 10.1017/S135561770410413X 15327731

[B63] Słowiński P. Alderisio F. Zhai C. Shen Y. Tino P. Bortolon C.. (2017). Unravelling socio-motor biomarkers in schizophrenia. npj Schizophr. 3:8. 10.1038/s41537-016-0009-x 28560254 PMC5441525

[B64] Sonnenschein S. Gomes F. Grace A. (2020). Dysregulation of midbrain dopamine system and the pathophysiology of schizophrenia. Front. Psychiatry 11:613. 10.3389/fpsyt.2020.00613 32719622 PMC7350524

[B65] Takenoshita S. Terada S. Yoshida H. Yamaguchi M. Yabe M. Imai N.. (2019). Validation of Addenbrooke's cognitive examination III for detecting mild cognitive impairment and dementia in Japan. BMC Geriatr. 19, 1–8. 10.1186/s12877-019-1120-4 31035933 PMC6489204

[B66] Thoma P. Soria Bauser D. Suchan B. (2013). BESST (Bochum Emotional Stimulus Set)—a pilot validation study of a stimulus set containing emotional bodies and faces from frontal and averted views. Psychiatry Res. 209, 98–109. 10.1016/j.psychres.2012.11.012 23219103

[B67] Trémeau F. (2022). A review of emotion deficits in schizophrenia. Dialog. Clin. Neurosci. 8, 59–70. 10.31887/DCNS.2006.8.1/ftremeau 16640115 PMC3181757

[B68] van der Schalk J. Hawk S. Fischer A. Doosje B. (2011). Moving faces, looking places: validation of the Amsterdam Dynamic Facial Expression Set (ADFES). Emotion 11, 907–920. 10.1037/a0023853 21859206

[B69] Vannuscorps G. Andres M. Caramazza A. (2020). Efficient recognition of facial expressions does not require motor simulation. Elife 9:e54687. 10.7554/eLife.54687.sa2 32364498 PMC7217693

[B70] Walther S. van Harten P. N. Waddington J. L. Cuesta M. J. Peralta V. Dupin L.. (2020). Movement disorder and sensorimotor abnormalities in schizophrenia and other psychoses - European consensus on assessment and perspectives. Eur. Neuropsychopharmacol. 38, 25–39. 10.1016/j.euroneuro.2020.07.003 32713718

[B71] Wang P. Gorenstein C. (2013). Psychometric properties of the Beck Depression Inventory-II: a comprehensive review. Braz. J. Psychiatry 35, 416–431. 10.1590/1516-4446-2012-1048 24402217

[B72] Wieser M. Brosch T. (2012). Faces in context: a review and systematization of contextual influences on affective face processing. Front. Psychol. 3:471. 10.3389/fpsyg.2012.00471 23130011 PMC3487423

[B73] Winkielman P. Niedenthal M. Oberman L. (2008). “The embodied emotional mind,” in Embodied Grounding: Social, Cognitive, Affective, and Neuroscientific Approaches, eds G. R. Semin, and E. R. Smith (Cambridge: Cambridge University Press), 263–288.

[B74] Wood A. Rychlowska M. Korb S. Niedenthal P. (2016). Fashioning the face: sensorimotor simulation contributes to facial expression recognition. Trends Cogn. Sci. 20, 227–240. 10.1016/j.tics.2015.12.010 26876363

