# Supplementary material for: Perception of emotionally incongruent cues: evidence for overreliance on body vs. face expressions in Parkinson's disease
Source: Front Psychol. 2024 May 6;15:1287952. doi: 10.3389/fpsyg.2024.1287952 (PMC11103677; doi:10.3389/fpsyg.2024.1287952)
Supplement: Supplementary file 1 [file Table_1.DOCX]

Perception of emotionally incongruent cues: Evidence for overreliance on body vs. face expressions in Parkinson’s disease

**Supplemental Information**

**Group differences in emotion perception of incongruent composites: full breakdown by the face emotion.**

A 4 (facial emotion: happiness, sadness, anger, fear) × 3 (categorization tendency: as face, as body, other) × 3 (group: PD, SZ, HC) mixed ANOVA was conducted.

Significant effects were found for categorization tendency, *F* (2, 226) = 139.35, *p* < 0.001*,* ƞ^2^*_p_* = .55. Significant interaction effects were found for categorization tendency × group, *F* (4, 226) = 5.64, *p* < 0.001*,* ƞ^2^*_p_* = .09, and facial emotion × categorization tendency, *F* (6, 678) = 106.53, *p* < 0.001*,* ƞ^2^*_p_* = .49. Importantly, the three-way interaction of facial expression × categorization tendency × age, *F* (6, 678) = 9.05, *p* < 0.001*,* ƞ^2^*_p_* = .14, was significant.

In order to better understand the 3-way interaction, we first conducted separate mixed ANOVAs for each facial expression using a 3 (categorization tendency: as face, as body, other) × 3 (group: PD, SZ, HC), followed by t-tests to examine the origin of interactions (see Figure S1).

Happy faces

Significant effects were found for categorization tendency, *F* (2, 226) =263.77, *p <* 0.001*,* ƞ^2^*_p_* = .7, age, *F* (1, 52) = 16.27, *p* < 0.001*,* ƞ^2^*_p_* = .24 and categorization tendency × age interaction, *F* (4, 226) = 17.47, *p* < 0.001*,* ƞ^2^*_p_* = .24. Compared to individuals with PD, SZ and HC tended to categorize incongruent composites as the face (*p*<.001). Individuals with PD were more likely to categorize them as the body emotion or other emotion (*p*<.001)

Sad faces

Significant effects were found for categorization tendency*, F* (2, 226) = 119.13, *p* <.001*,* ƞ^2^*_p_* = .51, and for the categorization tendency × age interaction, *F* (4, 226) = 5.04, *p* =.001*,* ƞ^2^*_p_* = .08. Individuals with SZ were more likely to categorize composites as the face compared to PD group (*p*=.01), while they were less likely to categorize them as bodily expressions compared to PD (*p*=.001) and HC (*p*=.02).

Angry faces

Significant effects were found for the categorization tendency, *F* (2, 226) = 60.02, *p* < 0.001*,* ƞ^2^*_p_* = .35 and for the categorization tendency × age interaction, *F* (4, 226) = 2.81, *p* =.03*,* ƞ^2^*_p_* = .05. Following comparisons revealed that individuals with SZ tended to categorize the incongruent composites as other emotion (*p*<.001).

Fearful faces

Significant effects were found for categorization tendency, *F* (2, 226) = 36.06, *p* < 0.001*,* ƞ^2^*_p_* = .24, and the categorization tendency × age interaction, *F* (4, 226) = 4.68, *p* =.001*,* ƞ^2^*_p_* = .08. Individuals with SZ were significantly more likely to categorize the incongruent composites as other compared to HC (*p*=.005) and PD (*p*<.001).

*
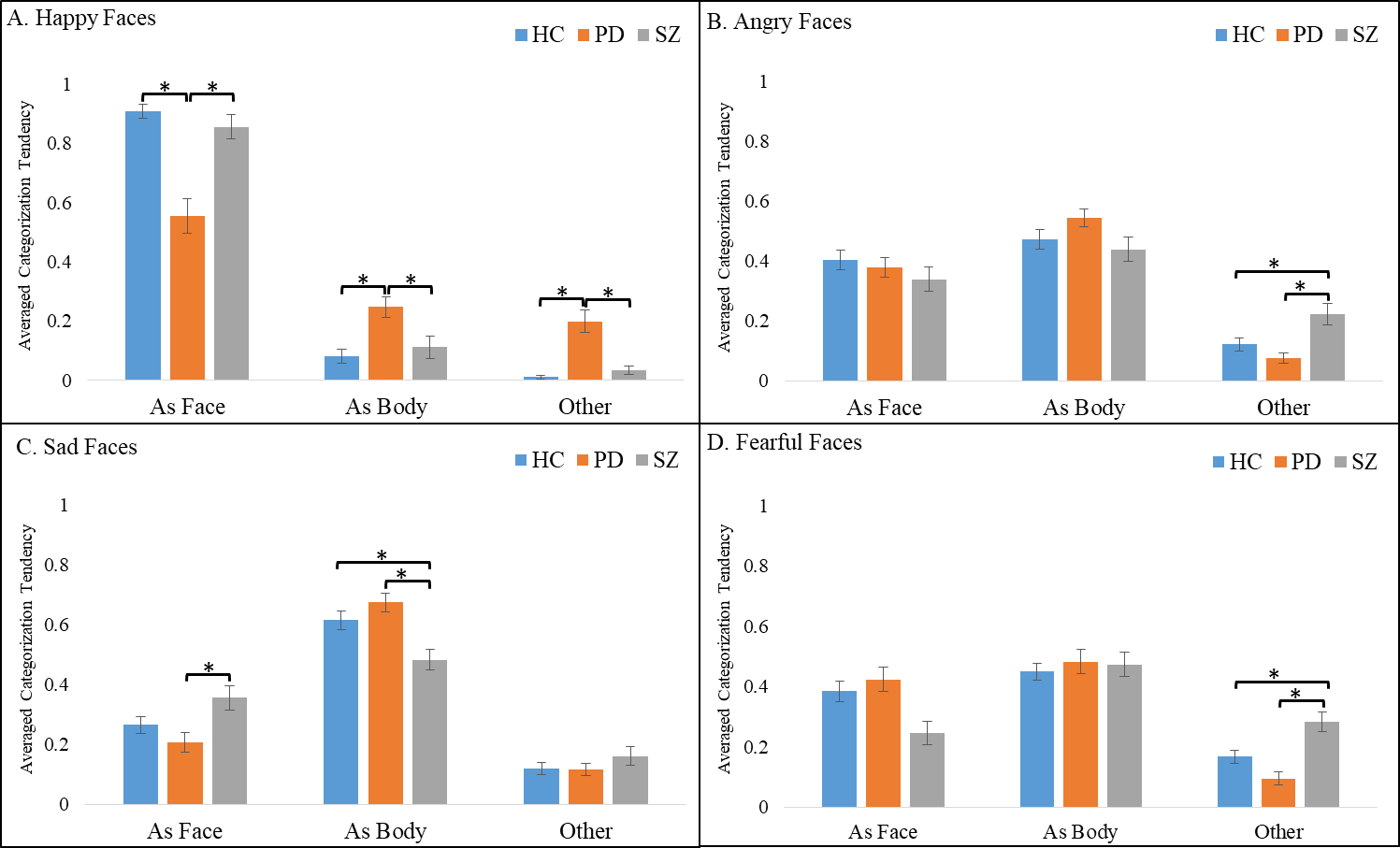
Figure S1*. The averaged categorization tendency of: (A) happy faces, (B) angry faces, (C) sad faces and (D) fearful faces appearing with incongruent bodies for the HC, PD and SZ groups. The categorizations: as face emotion, as body emotion and other (i.e., the chosen emotion did not correspond to the face or the body) are placed along the x-axis. Error bars represent standard errors.

* *p* < 0.01 . HC = healthy controls, PD = individuals with Parkinson’s disease, SZ = individuals with schizophrenia.

**Group differences in emotion perception: isolated faces, isolated bodies, and emotionally congruent faces with bodies.**

A 3 (group: PD, SZ, HC) × 3 (cue: face, body, face with body) × 4 (emotions: happiness, anger, sadness, fear) mixed ANOVA was run on the mean affective perception per cue for each emotion


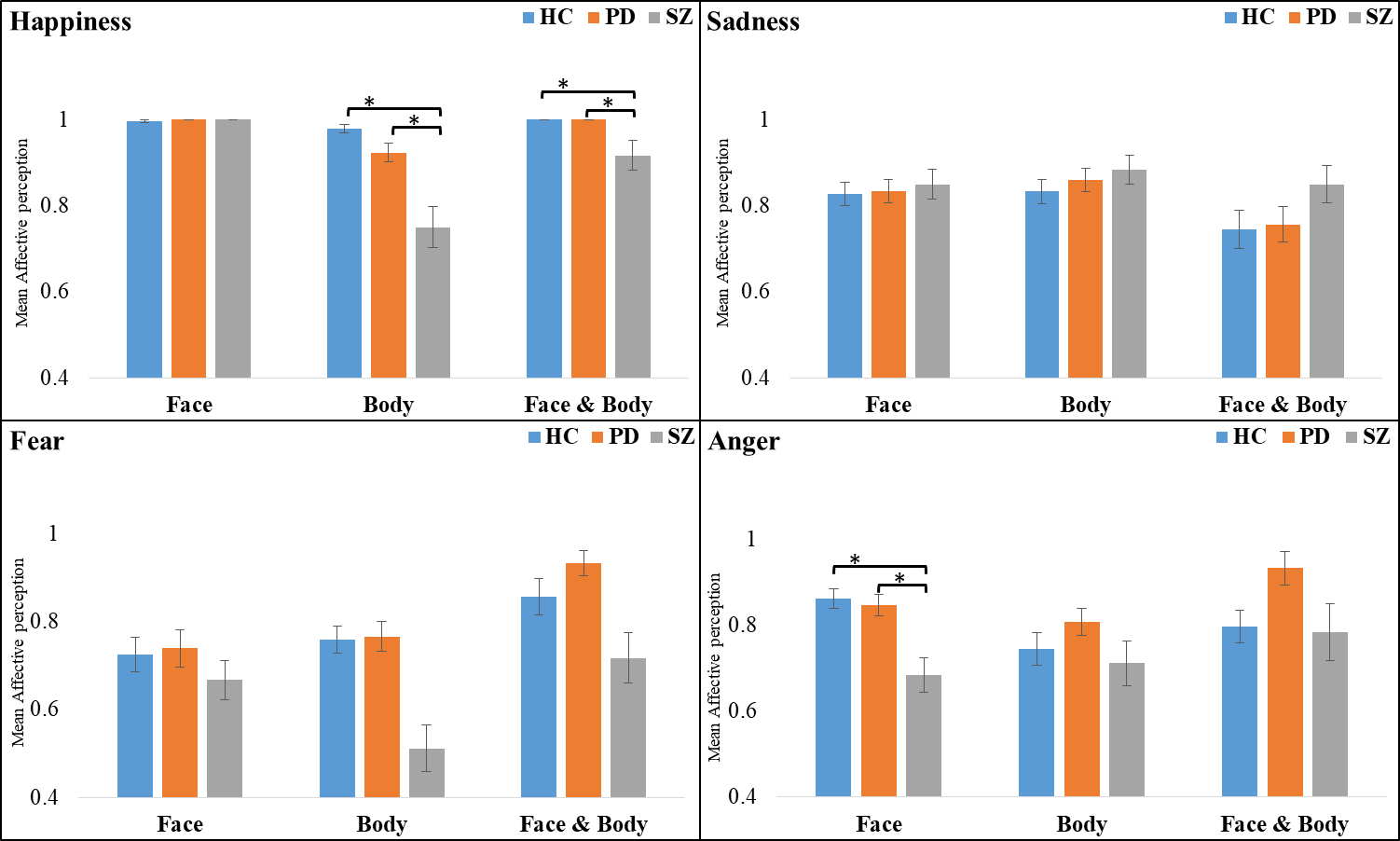
The results reveal significant main effects for cue, *F* (2, 226) = 12.05, *p* < .001, ƞ^2^*_p_* =.10, indicating that bodily emotions gained the lowest mean affective perception while facial expressions and congruent faces with bodies showed comparable means (*p*<0.01, *p*=.28 respectively). For the emotions, *F* (3, 339) = 53.09, *p* < .001, ƞ^2^*_p_* =.32, happiness showed the highest affective perception average while fear gained the lowest mean (all *p*-values<0.01). Finally, a group main effect was found, *F* (2, 113) = 6.56, *p* = .002, ƞ^2^*_p_* =.10 indicating that SZ group showed deteriorated affective perception abilities compared to HC and PD, that showed comparable performance (*p*<0.05, *p*=.94 respectively).

*Figure S2*. Mean emotion perception of emotions by cue: face, body and emotionally congruent faces with bodies for the HC, PD and SZ groups. Error bars represents standard errors. ^*^ *p* < .01. HC = healthy controls, PD = individuals with Parkinson’s disease, SZ = individuals with schizophrenia.

Significant interaction effects were found for cue × group, *F* (4, 226) = 2.54, *p* = .04, ƞ^2^*_p_* =.04, cue × emotions, *F* (6, 678) = 11.32, *p* < .001, ƞ^2^*_p_* =.09; emotion × group, *F* (6, 339) = 6.26, *p* < .001, ƞ^2^*_p_* =.10; as well as for the three-way interaction, cue × emotion × group, *F* (12, 678) = 2.86, *p* = .001, ƞ^2^*_p_* =.05.

To break down the three-way interaction, 3 (group — PD, SZ, HC) × 3 (cue — face, body, face with body) mixed ANOVAs were performed separately for each emotion (happiness, anger, sadness, fear). Results are shown in Table S1.

| Table S1*. Summary of Mixed Analysis of Variance (ANOVA) for 3 (groups — PD, SZ and HC) × 3 (cue — face, body and face with body) separately for each emotion.* | | | | | |
| --- | --- | --- | --- | --- | --- |
| **Cue** | **Effect** | ***df*** | **F** | ***P*** | **ƞ^2^*_p_*** |
| Happiness | Group | (2, 113) | 22.70 | <.001 | .26 |
|  | Cue | )2, 226( | 38.75 | <.001 | .29 |
|  | Cue × Group | (4,226) | 12.84 | <.001 | .19 |
| Anger | Group | (2, 113) | 4.86 | .01 | .08 |
|  | Cue | )2, 226( | 4.74 | .01 | .04 |
|  | Cue × Group | (4,226) | 2.49 | .04 | .04 |
| Sadness | Group | (2, 113) | 1.33 | .27 | -- |
|  | Cue | )2, 226( | 5.05 | .01 | .04 |
|  | Cue × Group | (4,226) | .63 | .65 | -- |
| Fear | Group | (2, 113) | 9.61 | <.001 | .15 |
|  | Cue | )2, 226( | 15.79 | <.001 | .12 |
|  | Cue × Group | (4,226) | 2.31 | .06 | -- |

** p* < 0.05. ACE = Addenbrooke's Cognitive Examination; MMSE= Minimental test; FAB = Frontal Assessment Battery; MOCA=Montreal Cognitive Assessment; BDI = Beck Depression Inventory

Individuals with SZ were found to perform more poorly in perceiving anger from isolated facial expressions compared to individuals with PD (*p*=.001) and the HC groups (*p*<.001). The SZ group showed similar discrepancies in the perception of happiness from isolated body expressions and from combined faces with bodies (all *p*-values < .01).

| Table S2.  *Pearson’s correlations between the incongruent faces with bodies averaged categorization tendency (i.e. as face, as body and other), cognitive screening tests, BDI, motor UPDRS and education years.* | | | | | | | | | | |
| --- | --- | --- | --- | --- | --- | --- | --- | --- | --- | --- |
|  |  | ACE | FAB | BDI | MOCA | Education years | UPDRS Motor | | UPDRS Face | |
| HC | As Face | .16 | .04 | *.03* | .27 | .04 |  | |  | |
|  | As Body | .003 | .09 | .03 | -.13 | -.06 |  |  | |  |
|  | Other | -.32* | -.26 | .004 | -.29* | .04 |  | |  | |
| PD | As Face | .17 | .20 | .08 | .08 | .25 | -.09 | | .08 | |
|  | As Body | -.16 | -.08 | .04 | .02 | .06 | -.12 | | -.33 | |
|  | Other | .03 | -.25 | -.22 | -.13 | .38 | .38* | | .32 | |
| SZ | As Face | .14 | .05 | .11 | .03 | -.16 | .02 | | -.13 | |
|  | As Body | .06 | .23 | .25 | .23 | .14 | -.19 | | -.20 | |
|  | Other | -.28 | -.4* | -.18 | -.37* | .03 | .24 | | .17 | |

** p* < 0.05, ** *p* < 0.01. ACE = Addenbrooke's Cognitive Examination; MMSE= Minimental test; FAB = Frontal Assessment Battery; MOCA=Montreal Cognitive Assessment; BDI = Beck Depression Inventory; UPDRS Motor= Unified Parkinson’s disease rating scale (items 8-31), UPDRS Face= Unified Parkinson’s disease rating scale of the facial expression (item 19).

| Table S3.  *Pearson’s correlations between the mean affective perception accuracy* *of isolated faces, isolated bodies and congruent faces with bodies (FB), cognitive screening tests, BDI, motor UPDRS and education years.* | | | | | | | | | |
| --- | --- | --- | --- | --- | --- | --- | --- | --- | --- |
|  |  | | ACE | FAB | BDI | MOCA | Education years | UPDRS Motor | UPDRS Face |
| HC | Face | | .27 | .38* | *.05* | *.28* | *.13* |  |  |
|  | Body | | .14 | .03 | .04 | .29* | .006 |  |  |
|  | Congruent FB | | .23 | .24 | .08 | .11 | -.01 |  |  |
| PD | Face | .29 | | -.12 | .16 | -.18 | .10 | -.07 | -.31 |
|  | Body | .31 | | .44* | -.002 | .41* | .26 | -.15 | -.25 |
|  | Congruent FB | .29 | | -.20 | 05. | .02 | -.28 | -.08 | -.28 |
| SZ | Face | .57** | | .50** | -.42* | .55** | .04 | -.43* | .25 |
|  | Body | .43* | | .43* | .05 | .43* | .07 | -.30 | -.04 |
|  | Congruent FB | .35 | | .42* | -.23 | .32 | -.08 | -.20 | -.07 |

** p* < 0.05, ** *p* < 0.01. ACE = Addenbrooke's Cognitive Examination; MMSE= Minimental test; FAB = Frontal Assessment Battery; MOCA=Montreal Cognitive Assessment; BDI = Beck Depression Inventory; UPDRS Motor= Unified Parkinson’s disease rating scale (items 8-31), UPDRS Face= Unified Parkinson’s disease rating scale of the facial expression (item 19).
